# Calycosin suppresses TGF-β-induced epithelial-to-mesenchymal transition and migration by upregulating BATF2 to target PAI-1 via the Wnt and PI3K/Akt signaling pathways in colorectal cancer cells

**DOI:** 10.1186/s13046-019-1243-7

**Published:** 2019-06-07

**Authors:** Qun Wang, Weijun Lu, Tao Yin, Li Lu

**Affiliations:** 1 Department of Hepatopancreatobiliary Surgery, Hubei Cancer Hospital, Wuhan, Hubei 430079 People’s Republic of China; 2 Department of Medical Oncology, Hubei Cancer Hospital, Tongji Medical College, Huazhong University of Science and Technology, Wuhan, Hubei 430079 People’s Republic of China; 3 Colorectal Cancer Clinical Research Center of Wuhan, Wuhan, Hubei 430079 People’s Republic of China; 4 Colorectal Cancer Clinical Research Center of Hubei Province, Wuhan, Hubei 430079 People’s Republic of China; 5 Department of Gastrointestinal Surgery, Hubei Cancer Hospital, Wuhan, Hubei 430079 People’s Republic of China

**Keywords:** BATF2, Calycosin, Cell migration, Colorectal cancer, PAI-1

## Abstract

**Objectives:**

To determine whether the upregulation of basic leucine zipper ATF-like transcription factor 2 (BATF2) by calycosin suppresses the growth and epithelial-to-mesenchymal transition (EMT) in human colorectal cancer (CRC) cells.

**Method:**

Cells were cultured and treated with different concentrations of calycosin for different periods of time. Protein and mRNA expression was determined by western blotting and quantitative PCR. Cell migration was assessed by Transwell experiments. Co-immunoprecipitation and luciferase assays were used to analyze the association between BATF2 and plasminogen activator inhibitor-1.

(PAI-1). Cell apoptosis was determined by flow cytometry; β-catenin cellular localization was visualized by immunofluorescent staining.

**Results:**

Calycosin up-regulated the expression of BATF2 via the signal transducer and activator of transcription 3 (STAT3) pathway, which was antagonized by transforming growth factor beta (TGF-β), calycosin promoted the cell apoptosis and growth inhibition via phosphoinositide 3-kinase (PI3K)/Akt pathway. TGF-β promoted cell growth, which was inhibited by calycosin regulating the expression of proliferating cell nuclear antigen (PCNA) via the phosphoinositide 3-kinase pathway. TGF-β suppressed expression of BAX via the phosphoinositide 3-kinase pathway but induced cell apoptosis .calycosin enhanced the effect of TGF-β on cell apoptosis,In addition, calycosin suppressed TGF-β-induced cell migration by increasing BATF2 to target PAI-1. TGF-β-induced EMT was inhibited by calycosin in human CRC LoVo and HCT116 cell lines via the Wnt signaling pathway.

**Conclusions:**

The induction of BATF2 by calycosin may be a feasible therapeutic option for CRC.

**Graphical Abstract:**

.
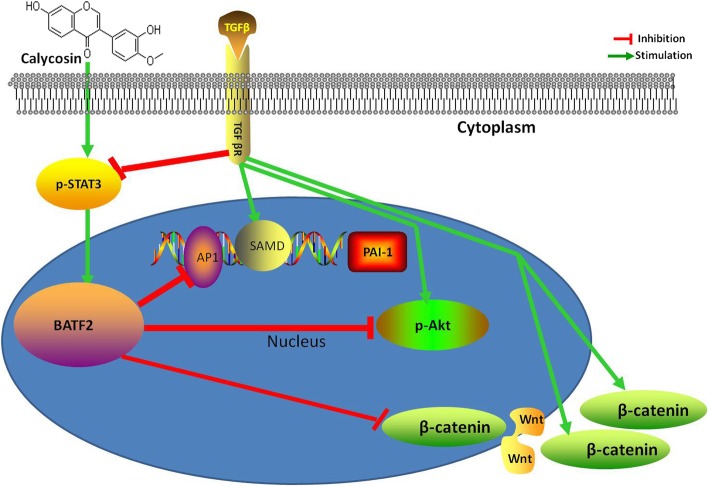

## Background

The basic leucine zipper (bZIP) ATF-like transcription factor (BATF) family [1] is a subgroup of the larger family of bZIP transcription factors, and its members belong to the AP-1 family of transcription factors. Functional analyses of BATF in cell culture systems and transgenic mice have demonstrated that it was a negative regulator of AP-1-mediated gene expression [2, 3], and cellular transformation by oncogenes that rely on robust AP-1 activity was blocked by the co-expression of BATF [2]. Recently, the induction of BATF2 was found to inhibit the hepatocyte growth factor (HGF)/MET signaling pathway [4] and to suppress angiogenesis and tumor growth by directly targeting ceruloplasmin via inhibition of the activity of the hypoxia inducible factor 1 alpha (HIF-1α)/vascular endothelial growth factor (VEGF) axis in colorectal cancer (CRC) cells [5].BATF2 regulates numerous cellular processes including growth inhibition and promotion of apoptosis [6, 7]. However, it’s role in the epithelial-to-mesenchymal transition (EMT) of CRC cells is unclear TGF-β signaling and activated Ras pathways have been implicated as key EMT inducers in CRC [8, 9], as localized CRC cells respond to TGF-β with growth inhibition and metastatic carcinoma cells proliferate after treatment with TGF-β [10–12]. Increased TGF-β levels within a primary tumor and high plasma levels of TGF-β correlate with a poor prognosis in patients with CRC [10, 11]. Wnt, phosphoinositide 3-kinase (PI3K)/Akt, and other signaling pathways may also play important roles in the EMT process during the progression of CRC [13–16]. Signal transducer and activator of transcription 3 (STAT3) is another important signaling pathway in the regulation of EMT in CRC. STAT3 interacts directly with Smad3 in vivo and in vitro, resulting in the attenuation of Smad3-Smad4 complex formation and suppression of Smad3 DNA binding to block TGF-β signaling [17]. BATF is a direct target of STAT3 [18]; thus, we were interested in determining the role of BATF2 in the STAT3-mediated inhibition of TGF-β-induced EMT in CRC.

We used the active components of the traditional Chinese medicine flavonoid calycosin (C_16_H_12_O_5_) to up-regulate BATF2 expression, and analyzed it’s effects on cell growth, apoptosis, migration, and EMT in CRC. The results showed that calycosin up-regulated BATF2 expression. This effect was inhibited by TGF-β via the STAT3 signaling pathway, which resulted in the inhibition of cell growth and the promotion of apoptosis through the PI3K pathway by Akt phosphorylation. Calycosin blocked TGF-β-induced migration and EMT by altering the expression of plasminogen activator inhibitor-1 (PAI-1) via the Wnt signaling pathway in LoVo and HCT116 human CRC cells. The results of this study suggested that the up-regulation of BATF2 by calycosin may be a therapeutic option for CRC.

## Materials and methods

### Cell culture

HCT116 and LoVo human CRC cell lines were obtained from Wuhan HealthCare Biotechnology Company (Wuhan, China). Cells were maintained in RPMI 1640 medium with 10% fetal bovine serum and incubated at 37 °C with 5% CO_2_ in a humidified atmosphere. Lipofectamine® RNAiMAX (Invitrogen, Carlsbad, CA), First Strand cDNA Synthesis Kit (TaKaRa, Dalian, China), and LY294002 (Promega, Fitchburg, WI) were used on the cells.

### MTT cell viability assay

The anti-proliferation effects of calycosin against tumor cells were evaluated by an MTT cell viability assay. Briefly, the cells were cultured in 96-well plates (5.0 × 10^3^ cells/well) for 12 h, and then incubated with various concentrations of calycosin (0, 50, 100, 150 μM, Phytomarker Ltd., Tianjin, China). After 6, 12, 24, and 48 h, cell viability was analyzed. The cells were treated with phosphate-buffered saline (PBS), LY294002, or LY294002 with TGF-β or calycosin. Optical density was read at 490 nm using a microplate reader (Bio-Tek Instruments, Winooski, VT). Cells were counted and a growth curve was generated.

### Quantitative PCR

Total RNA was extracted using the TRIzol reagent (Invitrogen), and first-strand cDNA was synthesized using M-MLV reverse transcriptase (TaKaRa). Quantitative PCR was performed in triplicate with SYBRII qPCR Master Mix according to the manufacturer’s protocol, using glyceraldehyde-3-phosphate dehydrogenase (GAPDH) as a control. The relative mRNA levels of the target genes were calculated with the 2^−ΔΔCt^ method.

### Western blot analysis

Total proteins were extracted from the cells and the concentrations were determined using a Bradford Protein Assay Kit (Beyotime Biotechnology, Beijing, China). The proteins (70 μg/lane) were separated on 10% sodium dodecyl sulfate-polyacrylamide gels and transferred to polyvinylidene difluoride membranes (Pierce, Waltham, MA). After blocking in 5% fat-free dry milk in Tris-buffered saline containing 0.1% Tween for 2 h, the membranes were incubated separately overnight at 4 °C with 1:1000 dilutions of primary antibodies against BATF2, PAI-1, GAPDH, phosphorylated (p)-STAT3, BCL2-associated X (BAX), Akt, p-Akt, STAT3, and proliferating cell nuclear antigen (PCNA) (all from bioswamp Biotechnology, China). After washing, the membranes were incubated separately with corresponding horseradish peroxidase-conjugated secondary antibodies (Zhongshan Biotechnology, Beijing, China) for 1 h at room temperature. Subsequently, the membranes were washed and the signals were visualized with SuperSignal West Dura Extended Duration Substrate.

### Cell migration assay

For migration assays, 3.0 × 10^4^ cells were seeded into the upper chamber of an 8-μM pore Transwell in 100 μL Dulbecco’s modified Eagle’s medium containing 0.2% bovine serum albumin. Cells were allowed to migrate for 5 h through the medium, 12 h through collagen, and 21 h through Matrigel. The migrated cells were fixed, stained, and counted in six random fields and averaged. The experiments were repeated three times.

### Co-immunoprecipitation assay

For the co-immunoprecipitation assay, LoVo cells were divided into two groups. Cell lysates were made from one group for subsequent western blot analysis. The remaining group was cultured with PBS (control), 100 μM calycosin, or 20 ng/mL TGF-β and 100 μM calycosin for 48 h, after which the cells were lysed. After prewashing with lysis buffer, 10 μL Protein A agarose beads was incubated with anti-BATF2 or IgG antibodies at room temperature for 10 min. The beads were mixed with the cell lysate overnight at 4 °C. The complex was eluted using an elution buffer (glycine HCl, pH 3.0), followed by heating for 10 min at 70 °C.

### Plasmid construction

The DNA fragment encoding the BATF2 gene was amplified from human cDNA with the following primers: BATF2 forward CTAGCTAGCGATGGATTGTGCCTC; and reverse CGGAATTCTGTTAGAAGTGGACTTG; the bold font indicates where the HindIII and XhoI cloning sites were introduced. The cDNA fragment obtained was verified by sequencing and cloned into pEGFP-N1 between the HindIII and XhoI sites to obtain pEGFP-N1-BATF2. The wild-type (WT) DNA fragment containing part of the promoter region (− 627 to − 636 from the transcription initiation site) of the PAI-1 gene was amplified from human genomic DNA with the following primers: forward GTAGCTAGCTAGCACAGAGAGAGTCTGGACAC; and reverse CCCTCGAGGGGCAGTCACCCCTAGGGCA. The DNA fragment obtained was cloned directly into the pGL3-Basic vector (Promega) between the *Kpn*I and *Bgl*II sites to obtain pGL3-PAI-1-WT. A mutant (MT) DNA sequence of the PAI-1 promoter region encompassing one putative BATF2 binding site (− 627 to − 636 from the transcription initiation site) was synthesized and inserted into the pGL3-Basic vector. The MT construct was designated pGL3-PAI-1-MT. Three small interfering RNAs (siRNAs) targeting PAI-1 (siPAI-1) were synthesized and constructed by Wuhan HealthCare Biotechnology Company (Wuhan, China). siPAI-1-3, with a silencing target sequence of 5′-GCTGACTTCACGAGTCTTT-3′, was the most transfection-efficient and was chosen for additional study. All plasmid constructs were confirmed by PCR.

### Luciferase reporter assays

Cells were grown at a density of 1.0 × 10^5^ cells/well in 6-well culture plates and transfected with plasmid DNA using Lipofectamine 2000 (Invitrogen) for 24 h. For luciferase assays, the cells were transiently co-transfected with the reporter gene construct (pEGFP-N1-BATF2), pGL3-PAI-1-MT, pGL3-PAI-1-WT, and pGL3-Basic plasmid encoding Renilla luciferase. The cells were incubated for 24 h and then mixed with PBS (control), 100 μM calycosin, 20 ng/mL TGF-β, or calycosin with TGF-β for 48 h. Firefly and Renilla luciferase activity was determined using the dual-luciferase reporter assay system (Promega).

### Flow cytometry

The cells were collected, washed with cold PBS, and suspended in 500 μl binding buffer. Then 5 μl Annexin V-FITC and 5 μl propidium iodide (Keygen, Nanjing, China) were added and mixed with the cells. After the incubation at room temperature for 15 min in the dark, the cells were subjected to flow cytometry analysis. The percentage of apoptotic cells was calculated as the sum of cells stained with both Annexin V and PI versus the cells stained only with Annexin V.

### Cytoimmunofluorescence staining

Different treated HCT116 and LoVo cells were seeded into 48-well plates for routine culturing. After washing in PBS, cells were fixed in 4% formaldehyde for 20 min at room temperature, treated with 0.5% Triton, and then blocked in 5% bovine serum albumin (BSA) at room temperature for 1 h. The cells were then incubated with goat anti-human β-catenin antibody (1100, bioswamp China) at at room temperature for 1 h and Anti-Rabbit IgG (1200, bioswamp) at 37 °C temperature in the dark for 1 h. After nuclear staining with 5 μg/ml DAPI for 1 min, cells were observed under an inverted microscope (DMIL LED). Negative control was performed by replacing the primary antibody with PBS.

### Statistical analysis

The results are expressed as the means ± standard deviations. Comparisons between multiple groups were made using a one-way analysis of variance (ANOVA), followed by Tukey’s post hoc test. Statistical analyses were conducted with The SPSS19.0 (SPSS Inc., Chicago, IL, USA). Significance was defined as *p* < 0.05.

## Results

### Calycosin inhibits growth in dose- and time-dependent manners

**Fig. 1:**
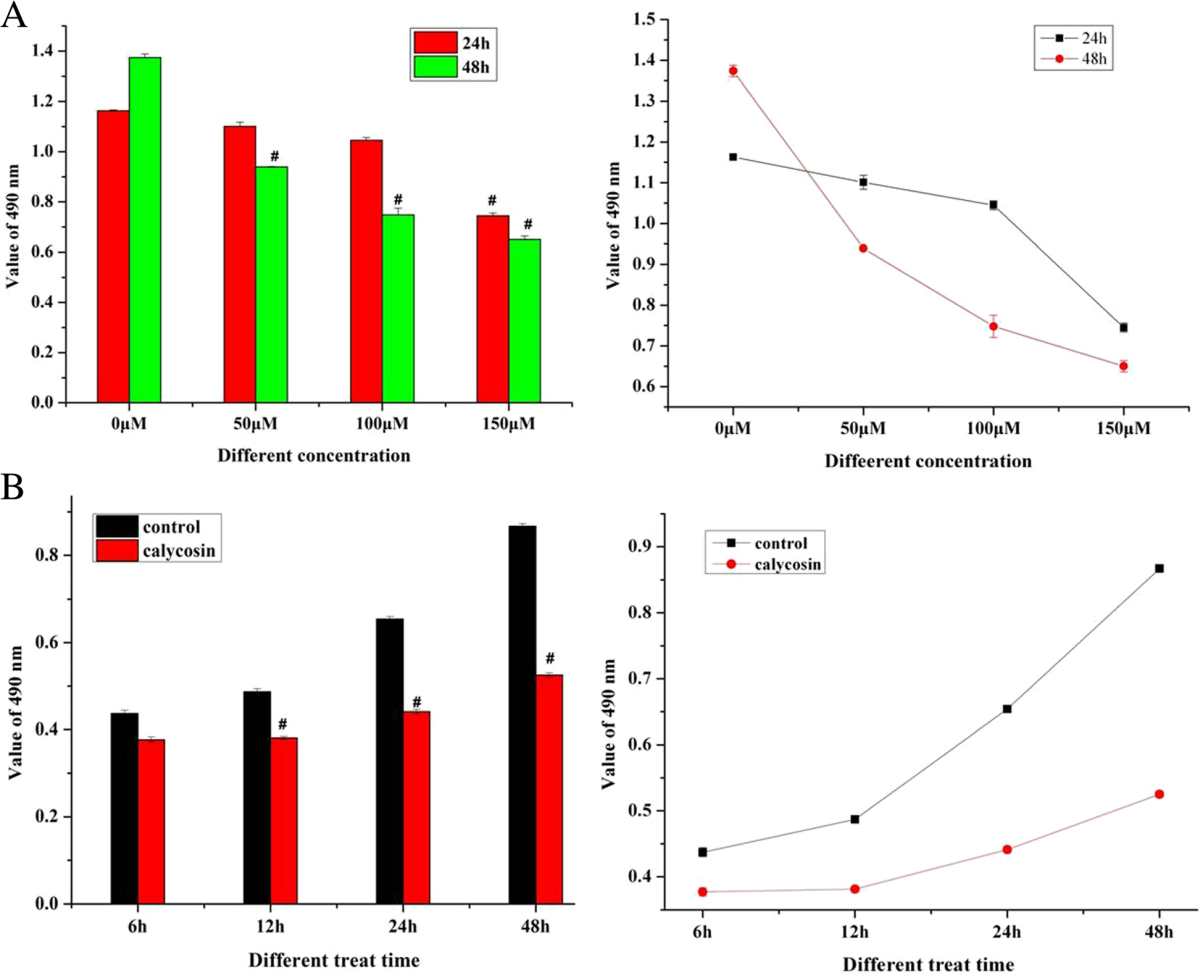
**a** Growth inhibition of CRC LoVo cells was significantly promoted by calycosin in a dose- and time-dependent manner. **b** Proliferation of LoVo cells was inhibited by treatment with 100 μM calycosin for 12 h

**Fig. 2:**
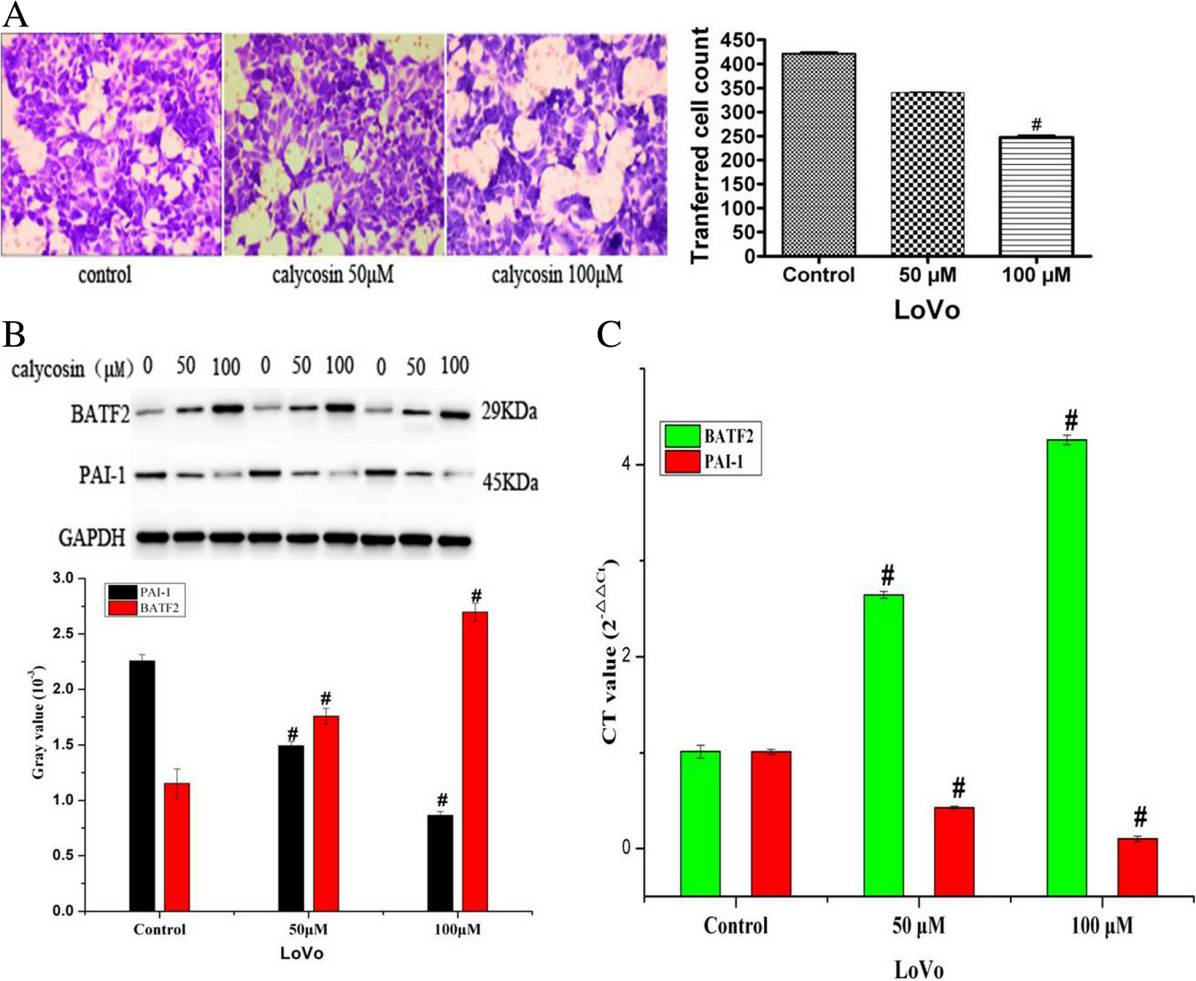
**a** Cell migration was significantly induced by 50 and 100 μM calycosin. **b** BATF2 and PAI-1 protein levels were evaluated by western blot analysis after LoVo cells were treated with 50 or 100 μM calycosin. **c** BATF2 and PAI-1 mRNA levels were determined with real-time PCR after LoVo cells were treated with 50 or 100 μM calycosin

As shown in Fig. 1a, calycosin inhibited the proliferation of LoVo cells in dose- and time dependent manners (Fig. 1a). This inhibition was significant at concentrations of 50 and 100 μM at 48 h or at a concentration of 150 μM at 12 or 48 h compared to control cells (*P* < 0.05). BATF2 over-expression was induced by treatment with 100 μM calycosin for 48 h (*P* < 0.01). Cell migration was inhibited significantly at 100 μM 48 h (Fig. 2a and b). On the basis of these results, we treated the cells with 100 μM calycosin (Fig. 1b) for 6, 12, and 48 h. LoVo cell growth in the treatment group was suppressed with 100 μM calycosin at 12, 24, and 48 h compared to the control group (*P* < 0.05). Thus, in the following experiments, the cells were treated with 100 μM calycosin for 48 h.

### Calycosin up-regulates BATF2 expression, which is inhibited by TGF-β via the STAT3 signaling pathway to promote growth inhibition involving Akt phosphorylation

**Fig. 3:**
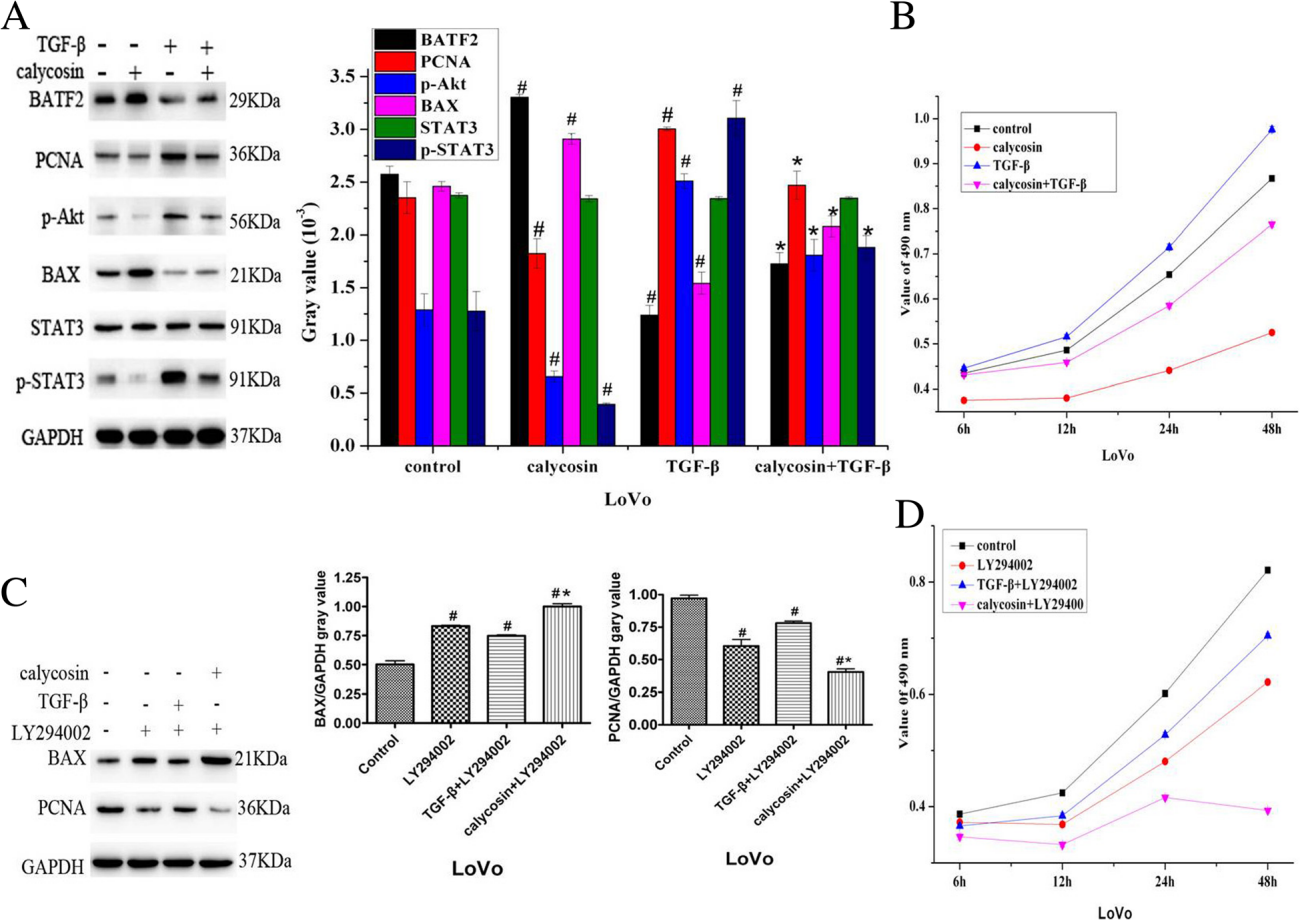
a BATF2, STAT3, p-STAT3, BAX, PCNA, and p-Akt protein levels were determined by western blot analysis after LoVo cells were treated with PBS, calycosin, TGF-β, or calycosin with TGF-β. **b** MTT assay was used to evaluate cell growth after LoVo cells were treated with PBS, calycosin, TGF-β, or calycosin with TGF-β. **c** BAX and PCNA protein levels were determined by western blot analysis after LoVo cells were treated with PBS, LY294002, or LY294002 with TGF-β or calycosin. **d** MTT assay was used to evaluate cell growth after LoVo cells were treated with PBS, LY294002, or LY294002 with TGF-β or calycosin after 6, 12, 24, and 48 h

To investigate the effects of calycosin- and TGF-β-induced BATF2 expression on cell growth and apoptosis, BATF2, STAT3, p-STAT3, BAX (marker of apoptosis), PCNA (marker of proliferation), and p-Akt levels were evaluated by western blotting. The expression of p-STAT3, which has binding sites in the BATF2 promoter [18], was increased by TGF-β and decreased by calycosin (*P* < 0.05). There was no significant difference in STAT3 expression (Fig. 3a). Cell growth was regulated by the PI3K signaling pathway activated by p-Akt, which was increased by TGF-β and decreased by calycosin (*P* < 0.05) (Fig. 3a). When calycosin and TGF-β were administered together, the up-regulation of PCNA, p-Akt, and p-STAT3 by TGF-β was inhibited (*P* < 0.05) (Fig. 3a); the TGF-β-induced down-regulation of BAX expression was blocked by calycosin (Fig. 3a) and LY294002 (Fig. 3c); the TGF-β-induced expression of PCNA was also blocked by calycosin (Fig. 3a) and LY294002 (Fig. 3c). Calycosin heightened the effect of LY294002. Compared to control cells, TGF-β promoted cell proliferation (*P* < 0.05) (Fig. 3b), and calycosin suppressed cell growth in a time-dependent manner (*P* < 0.05) (Fig. 3b). The promotion of cell growth induced by TGF-β was also effectively inhibited by calycosin (Fig. 3b), LY294002 (Fig. 3d), and calycosin with LY294002 (Fig. 3d) in a time-dependent manner (*P* < 0.05). The expression of BAX was increased by calycosin (Fig. 3c), LY294002 (PI3K signaling inhibitor), and calycosin with LY294002 (Fig. 3c). TGF-β induced suppression of BAX expression was reversed by LY294002 (Fig. 3c). Cell proliferation was assessed by PCNA protein expression, which was increased by TGF-β, but decreased by calycosin (*P* < 0.05) (Fig. 3d), LY294002, and calycosin with LY294002 (Fig. 3d).

### Calycosin enhanced the effect on TGF-β induced apoptosis

**Fig. 4:**
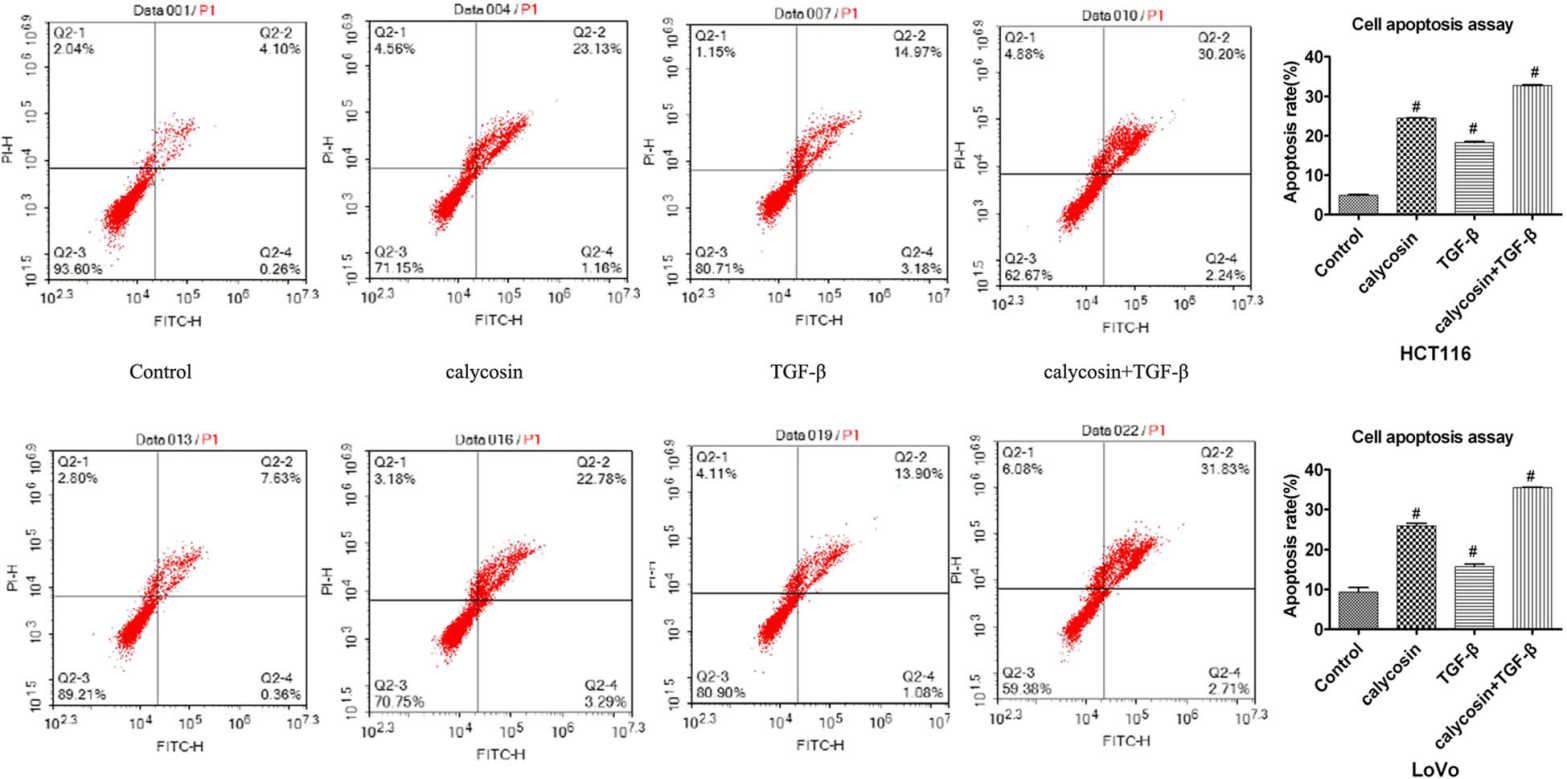
After HCT116 and LoVo treated with PBS (control), 100 μM calycosin, or 20 ng/mL TGF-β and 100 μM calycosin for 48 h cell apoptosis was determined by flow cytometry

TGF-β suppressed the expression of BAX that was recognized as maker of cell apoptosis and TGF-β induced suppression of BAX expression was reversed by LY294002 and calycosin respectively. But the role of TGF-β in cancer is complex and paradoxical,varying by cell type and stage of tumorigenesis. To identified the effect of TGF-β on LoVo and HCT116 cells, apoptosis was determined by flow cytometry after LoVo and HCT116 cells were treated with PBS (Control), TGF-β20ng/ml,calycosin100μM and TGF-β combination with calycosin for 48 h. As it was shown in Fig. 4, compared to the control, TGF-β and calycosin promoted cell apoptosis respectively in both LoVo and HCT116 cell (*P* < 0.05). and cell apoptosis rates were up-regulated to highest in combination group of TGF-β and calycosin.

### Calycosin inhibits cell migration and regulates BATF2 and PAI-1 expression in a dose-dependent manner

To determine the relationship between the inhibition of cell migration and the expression of BATF2 and PAI-1, we evaluated the mRNA and protein levels of BATF2 and PAI-1, as their expression has been confirmed in distant metastases in human CRC. After culturing with 50 or 100 μM calycosin for 48 h, cell migration was inhibited in a dose-dependent manner. The number of migrated cells was decreased after treatment with 50 μM calycosin for 48 h (*P* < 0.05) (Fig. 2a), and it was decreased further by treatment with 100 μM calycosin for 48 h (*P* < 0.01) (Fig. 2a). As shown in Fig. 2b and c, respectively, BATF2 protein and mRNA expression was increased by 50 μM (*P* < 0.05) and 100 μM (*P* < 0.01) calycosin. Conversely, PAI-1 protein and mRNA expression was decreased by 50 μM (*P* < 0.05) and 100 μM (*P* < 0.01) calycosin. Thus, calycosin inhibited cell migration in a dose-dependent manner and regulated BATF2 and PAI-1 expression.

### Calycosin attenuates TGF-β-induced cell migration by regulating PAI-1 mRNA and protein expression

**Fig. 5:**
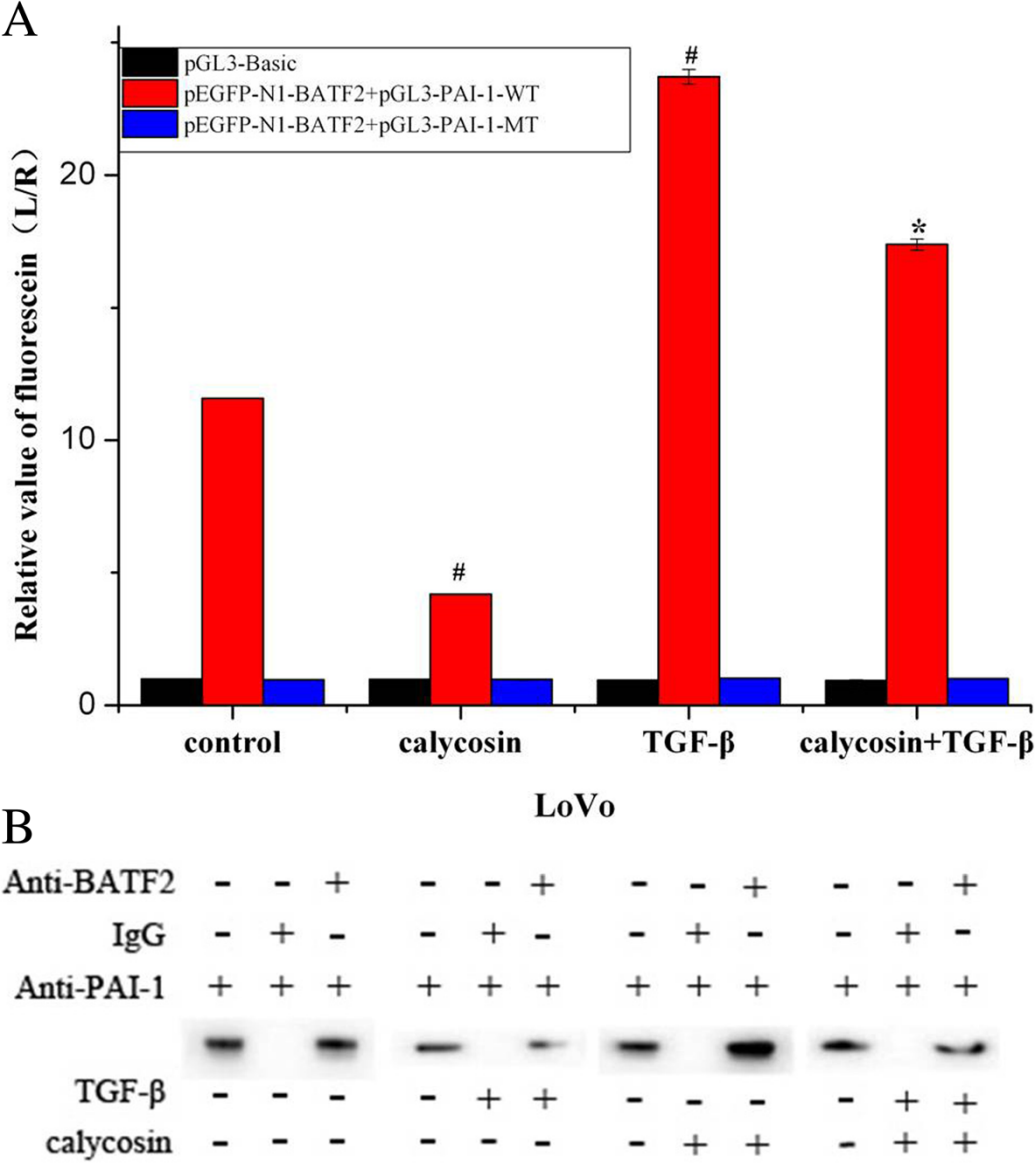
**a** Wild-type and mutant PAI-1 promoter activity was determined by luciferase assays after transfection of LoVo cells with pEGFP-N1-BATF2 and treatment with calycosin, TGF-β, or calycosin with TGF-β. **b** After LoVo cells were treated with calycosin, TGF-β, or calycosin with TGF-β, a co-immunoprecipitation assay was performed with anti-BATF2 or IgG antibodies and anti-PAI-1 antibodies

**Fig. 6:**
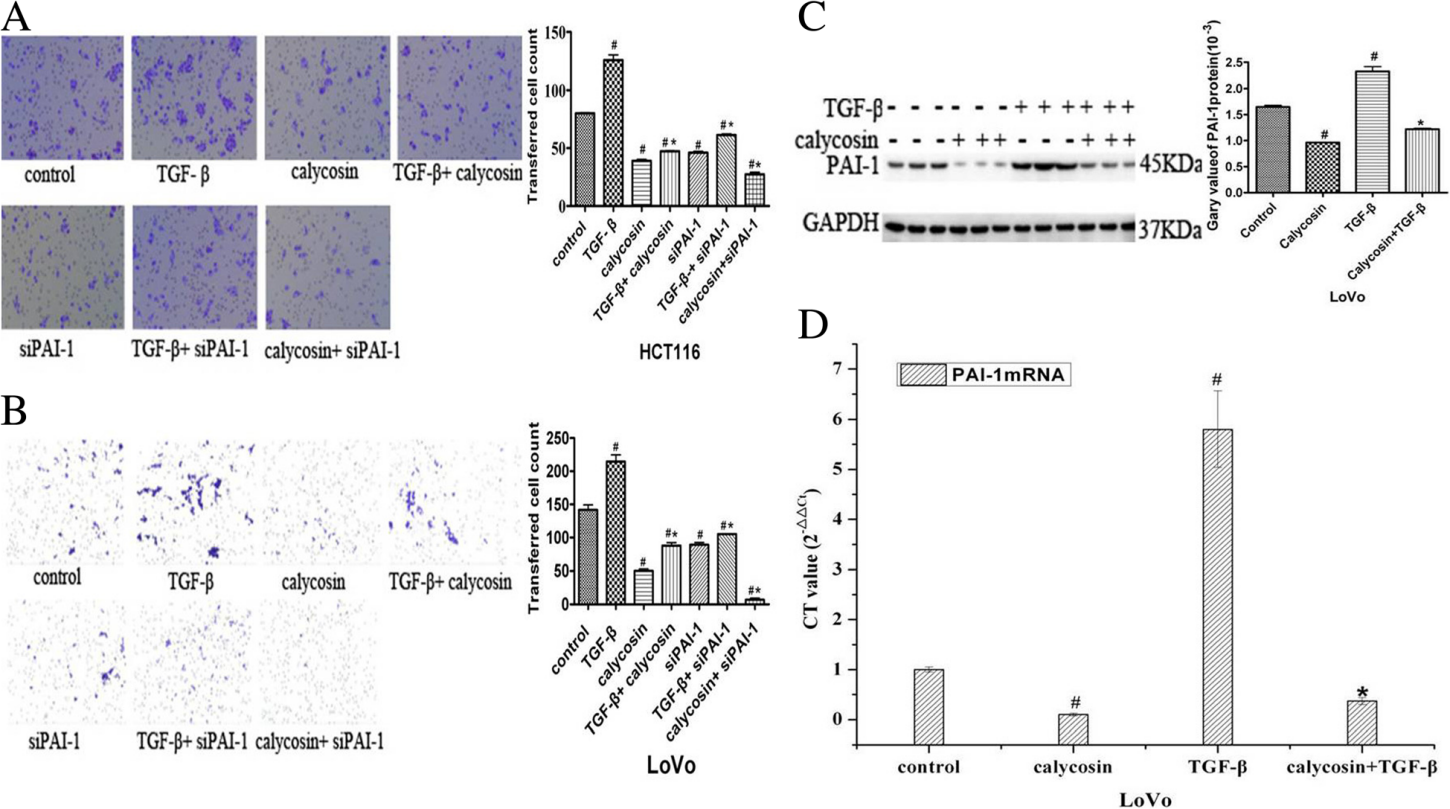
**a** HCT116 cell migration was assayed with Transwell experiments after the cells were treated with PBS, calycosin, TGF-β, siPAI-1, or siPAI-1 with TGF-β or calycosin. **b** LoVo cell migration was assayed with Transwell experiments after the cells were treated with PBS, calycosin, TGF-β, siPAI-1, or siPAI-1 with TGF-β or calycosin. **c** PAI-1 protein expression was determined by western blot analysis after LoVo cells were treated with PBS, calycosin, TGF-β, or calycosin with TGF-β. **d** PAI-1 mRNA expression was determined with real-time PCR after LoVo cells were treated with PBS, calycosin, TGF-β, or calycosin with TGF-β

Cell migration was suppressed by the calycosin-mediated downregulation of PAI-1, which is a target gene of TGF-β that induces promoter activity and endogenous expression confirmed by us (Fig. 5a and b). Thus, we presumed that TGF-β-induced cell migration would be blocked by calycosin and the siRNA-mediated downregulation of PAI-1. Highly invasive human CRC LoVo and HCT116 cells were cultured for 48 h with PBS (control), 100 μM calycosin, 20 ng/mL TGF-β, calycosin with TGF-β, siPAI-1, TGF-β with siPAI-1, or calycosin with siPAI-1. Cell migration was assessed with Transwell experiments. LoVo and HCT116 cell migration was promoted by TGF-β (*P* < 0.05) (Fig. 6a and b) and suppressed by calycosin, siPAI-1, and calycosin with siPAI-1 (*P* < 0.05) (Fig. 6a and b). TGF-β-induced cell migration was decreased by calycosin, siPAI-1, and calycosin with siPAI-1 (Fig. 6a and b). In LoVo cells, PAI-1 protein and mRNA expression was up-regulated by TGF-β (*P* < 0.05) and down-regulated by calycosin (Fig. 6c, d; *P* < 0.05). The upregulation of PAI-1 by TGF-β was completely suppressed by calycosin. The TGF-β-induced inhibition of cell migration caused by calycosin was in accordance with the TGF-β-induced inhibition of PAI-1 expression caused by calycosin.

### Direct targeting of PAI-1 by BATF2 is regulated by Calycosin and TGF-β in an antagonistic manner

To demonstrate the relationship between BATF2 and PAI-1 at the pre- and post-transcriptional levels, we performed luciferase assays to determine wild-type and mutant PAI-1 promoter activity induced by pEGFP-N1-BATF2 in LoVo cells after treatment with calycosin, TGF-β, or calycosin with TGF-β. The differentially treated CRC cells were collected and transfected with the plasmid constructs, and no significant fluorescence was observed in the blank control (pGL3-Basic) and mutant control plasmid (pGL3-PAI-1-MT) groups. Compared to the pGL3-Basic and pGL3-PAI-1-MT plasmids, there was an increase of fluorescence from the pGL3-PAI-1-WT plasmid (*P* < 0.05) after the pEGFP-N1-BATF2 reporter construct was transfected into the cells. Luciferase assays in the control group indicated that the direct action of BATF2 on PAI-1 was at the pre-transcriptional level. Wild-type PAI-1 promoter activity was down-regulated by calycosin (*P* < 0.05) and up-regulated by TGF-β (*P* < 0.05). The promotion of TGF-β-induced PAI-1 promoter activity was partially blocked by calycosin (*P* < 0.05) (Fig. 5a). A co-immunoprecipitation assay confirmed the interaction of BATF2 and PAI-1 at the post-transcriptional level. Calycosin promoted the effect of BATF2 on PAI-1 (Fig. 5b), whereas TGF-β inhibited this effect. Calycosin attenuated the inhibitory effects of TGF-β on the interaction between BATF2 and PAI-1.

### Calycosin blocks TGF-β-induced EMT via β-catenin nuclear translocation

**Fig. 7:**
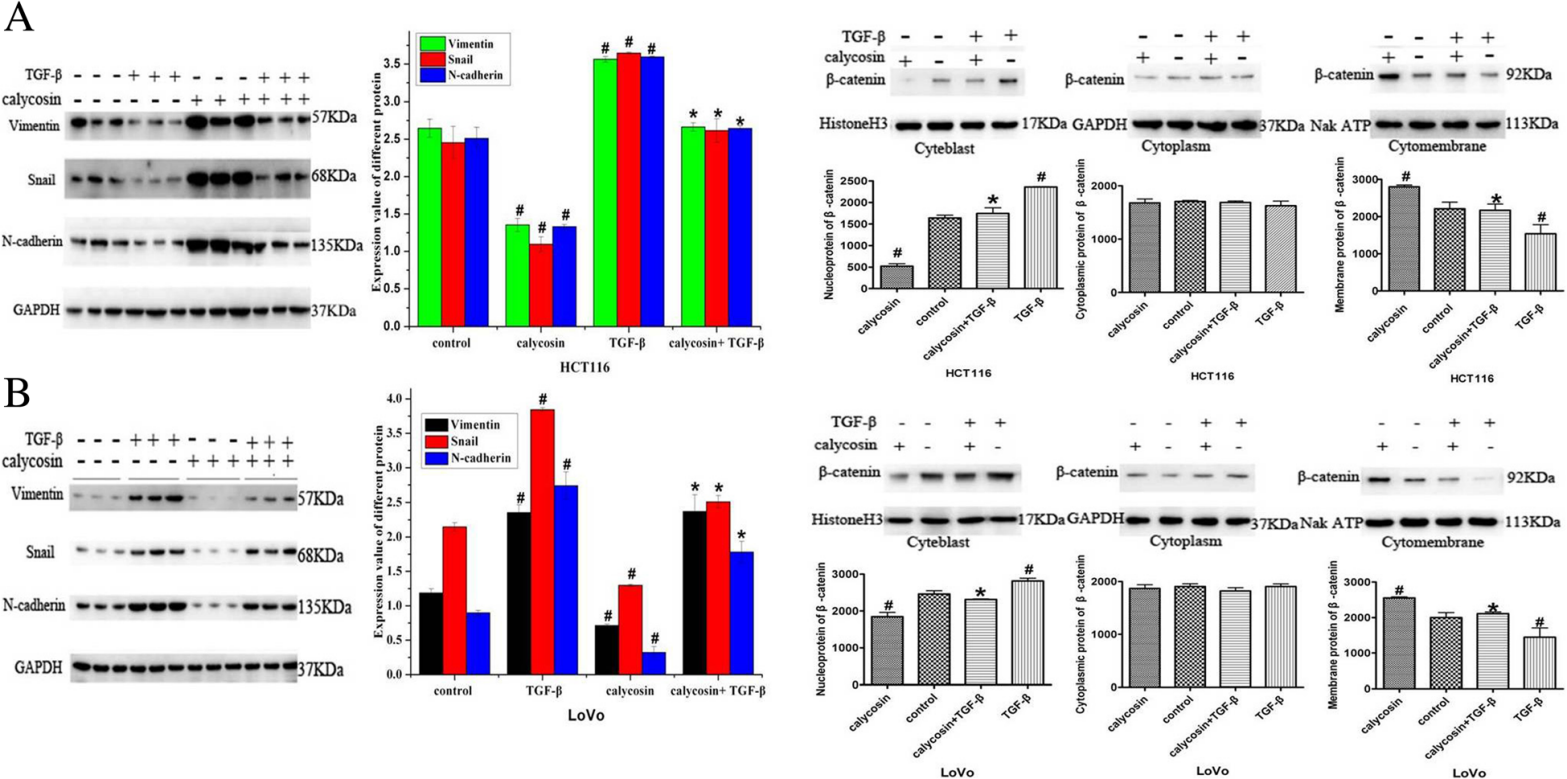
**a** After HCT116 cells were treated with calycosin, TGF-β, or calycosin with TGF-β, western blot analysis was used to determine the protein levels of vimentin, Snail, and N-cadherin and the subcellular distribution of β-catenin protein. **b** After LoVo cells were treated with calycosin, TGF-β, or calycosin with TGF-β, western blot analysis was used to determine the protein levels of vimentin, Snail, and N-cadherin and the subcellular distribution of β-catenin protein

To investigate the role of calycosin in TGF-β-induced EMT and the underlying mechanisms, westernblotting was used to detect the expression of vimentin, Snail, and N-cadherin, which function as negative regulators of EMT in CRC. The nucleus translocation of β-catenin was determined by using westernblotting to detect its expression in the cytoplasm and cytomembrane. Highly invasive human CRC LoVo and HCT116 cells were cultured with PBS (control), 100 μM calycosin, 20 ng/mL TGF-β, or calycosin with TGF-β for 48 h The expression of vimentin, Snail, N-cadherin was promoted by TGF-β (*P* < 0.05) and suppressed by calycosin (*P* < 0.05) in HCT116 (Fig. 7a) and LoVo cells (Fig. 7b). The TGF-β-induced upregulation of vimentin, Snail, and N-cadherin expression was blocked by calycosin in HCT116 cells (Fig. 7a) and attenuated by calycosin in LoVo cells (Fig. 7b) in the combined treatment group compared to the control group. Cytoblast β-catenin proteins were increased by TGF-β and decreased by calycosin (*P* < 0.05) in HCT116 (Fig. 7a) and LoVo cells (Fig. 7b). Conversely, β-catenin expression in the cytomembrane was decreased by TGF-β and increased by calycosin (*P* < 0.05) in HCT116 (Fig. 7a) and LoVo cells (Fig. 7b). In spite of cell location in cytomembrane and nucleus, the changes in β-catenin protein induced by TGF-β were blocked by calycosin (*P* < 0.05) (Fig. 7a, b).

### Calycosin retains TGF-β-induced β-catenin nucleus import

**Fig. 8:**
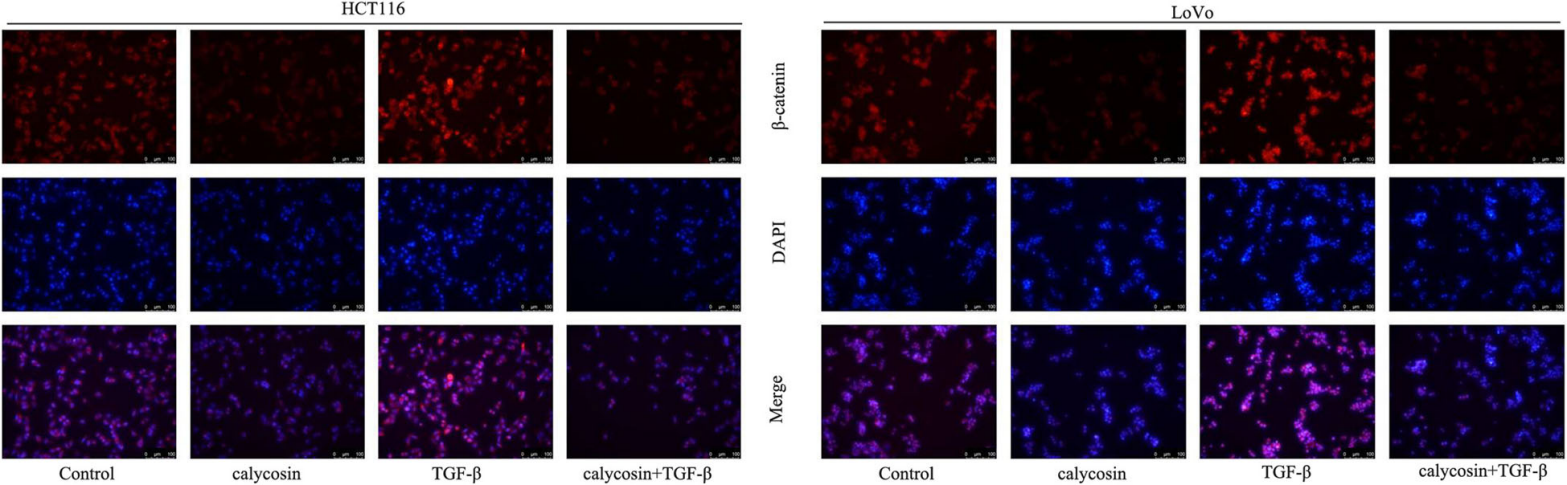
β-catenin cellular localization was visualized by immunofluorescent staining. HCT116 and LoVo cells were treated with PBS (control), 100 μM calycosin, or 20 ng/mL TGF-β and 100 μM calycosin for 48 h, following which they were fixed in methanol, incubated with the indicated antibodies, stained with Alexa Fluor 594-conjugated secondary antibodies and counterstained with propidiumiodide. Slides were then mounted and examined under a fluorescence microscope

To determine whether calycosin and TGF-β affect β-catenin localization in CRC cells, HCT116 and LoVo cells were heavily seeded, and β-catenin was analyzed by Cytoimmunofluorescence staining. Interestingly, β-catenin nuclear localization was clearly retained and induced by calycosin and TGF-β conversely (Fig. 8). Calycosin reversed TGF-β- induced nucleus importing of β-catenin in both HCT116 and LoVo CRC cells (Fig. 8).

## Discussion

Calycosin, is mainly bioactive compounds of isoflavones. It exhibit a variety of properties for alternative hormone therapy [19]. Calycosin was found able to play part alternative role of interferons and was considered as “plant interferon” in Chinese traditional medicine. Calycosin induces apoptosis and inhibits growth by reducing p-Akt levels and down-regulating microRNA-95 in CRC cells [7]. BATF2 expression has been induced by interferons via STAT1 [20] and by AG490 via STAT3 [21]. STAT3 signaling can be antagonized by STAT1 [22], and accumulating evidence has shown that the PI3K and STAT3 pathways can be activated by TGF-β [17, 23, 24]. We wandered that whether BATF2 could be induced by calycosin as interferons did. In our study, TGF-β simultaneously activated two signaling pathways: PI3K and STAT3, which were both inhibited by calycosin. BATF2 expression was down-regulated and up-regulated by TGF-β and calycosin, respectively, and TGF-β-reduced BATF2 expression could be rescued by calycosin. BATF2 is regulated by STAT3, which inhibits target gene promoter activity by promoting phosphorylation [18], and it has been demonstrated that the STAT signaling inhibitor AG490 up-regulates BATF2 mRNA expression, whereas the PI3K pathway inhibitor LY294002 has no such effect [22]. We successfully used calycosin to suppress TGF-β-induced-STAT3 phosphorylation. Thus, we believe that calycosin induces the upregulation of BATF2 expression antagonized by TGF-β by inactivating the STAT3 signaling pathway. BATF2 overexpression promotes growth inhibition and apoptosis in cancer cells [25]. We revealed that the TGF-β-induced regulation of PCNA and BAX expression and cell proliferation were repressed by an PI3K signaling inhibitor (LY294002) and calycosin, respectively, which exert their effects by inhibiting the phosphorylation of Akt. Furthermore calycosin heightened the effect of LY294002 on cell proliferation. We came to the conclusion that the TGF-β-induced promotion of growth blocked by calycosin involved the PI3K pathway via regulation of PCNA. We also uncovered that TGF-β suppressed expression of BAX via PI3K /Akt sailing but cell apoptosis was promoted by TGF-β, we presumed that it was maybe that apoptosis induced by TGF-β was determined by the way more than PI3K /Akt, because it was reported that the ratio of Smad3 to Akt correlated with the sensitivity of cells to TGF-β induced apoptosis [26, 27].

There is clinical evidence implicating PAI-1 as a key factor in tumor invasion and metastasis. PAI-1 is arguably one of the most well studied TGF-β-target genes, which is confirmed in the present study via luciferase reporter assays. However, the regulation of PAI-1 gene expression by TGF-β is not understood completely [28]. We discovered that when the PAI-1 gene was silenced with siRNA, cell migration and the TGF-β-induced migration of LoVo and HCT116 cells were significantly blocked. Calycosin inhibited cell migration by down-regulating PAI-1 and up-regulating BATF2 in a dose-dependent manner, and TGF-β-induced cell migration was also inhibited by calycosin in LoVo and HCT116 cells. Calycosin augmented the effect of siPAI-1 on the inhibition of cell migration. Both untreated and TGF-β-induced cell migration were suppressed by calycosin via the regulation of PAI-1 expression, which is associated with cell motility [29]. We demonstrated that the TGF-β-induced inhibition of cell migration caused by calycosin and siPAI-1 was in accordance with the TGF-β-induced inhibition of PAI-1 expression caused by calycosin and siPAI-1.Further to elucidate the mechanisms underlying the calycosin-mediated regulation of TGF-β signaling and PAI-1 and BATF2 expression, we performed luciferase reporter assays to confirm that TGF-β-induced PAI-1 promoter activity was inhibited by calycosin in accordance with the regulation of cell migration by calycosin. We also used co-immunoprecipitation assays to show that BATF2 regulated PAI-1, which was inhibited by TGF-β. Calycosin attenuated the inhibitory effects of TGF-β on the interaction of BATF2 with PAI-1. Thus, we conclude that TGF-β-induced cell migration can be suppressed by calycosin, which increases the expression of BATF2 to down-regulate PAI-1.

The progression of CRC to invasive and metastatic disease may involve the localized occurrence of EMT; the activation of EMT is considered a hallmark of metastasis in several human cancers. TGF-β strongly induces EMT, which contributes to the generation and accumulation of fibroblasts and myofibroblasts responsible for the excessive deposition of extracellular matrix. TGF-β signaling is a key EMT inducer in CRC [30], and we confirmed that TGF-β induced EMT in CRC cells by promoting the expression of vimentin, Snail, and N-cadherin, which negatively regulated EMT in CRC cells. We found that TGF-β-induced EMT could be reversed by calycosin to suppress the expression of vimentin, Snail, and N-cadherin in LoVo and HCT116 cells. To determine whether BATF2 was involved in TGF-β-induced EMT, we analyzed the subcellular localization of β-catenin after LoVo and HCT116 cells were treated with TGF-β, calycosin, or TGF-β combination with calycosin. TGF-β induced β-catenin localization from the cytomembrane to the cytoblast, and the TGF-β-induced nuclear accumulation of β-catenin was reversed by calycosin in HCT116 and LoVo cells. PI3K/Akt activation is believed to be essential for TGF-β1-induced EMT in tumor cell lines [31]. Ormanns et al [32] reported that activation of the PI3K pathway was not associated with nucleus β-catenin expression in CRC cells. They concluded that although the transcriptional activity of nucleus β-catenin depended on PI3K activity. PI3K its own did not affect the subcellular localization of β-catenin. The inhibition of Akt phosphorylation caused by calycosin may be partially responsible for TGF-β-induced EMT via inactivation of the PI3K pathway. It was BATF2 induced by the calycosin in the cell nucleus mediated prevention of the TGF-β-induced nucleus accumulation of β-catenin, which is considered to be a sign of the activation of the Wnt signaling pathway. β-catenin degradation occurs in both the cytoplasm and nucleus according to Wnt status. In the “Wnt-off” state, β-catenin is mainly degraded in the cytoplasm, whereas in the “Wnt-on” state, it is mainly degraded in the nucleus via the ubiquitin proteasome system [33]. In the present study, no difference was found in β-catenin cytoplasmic expression in all groups, regardless of treatment, and the TGF-β-induced nuclear translocation of β-catenin was blocked by calycosin Thus, it is clear that the nucleus, and not cytoplasmic, degradation of β-catenin is activated by calycosin, which causes a switch to the “Wnt-on” state. In this state, β-catenin translocates into the nucleus due to the disassembly of the β-catenin destruction complex. β-catenin ubiquitination in the nucleus is mainly mediated by c-Cbl and tripartite motif-containing protein 33 (TRIM33) [34, 35]. The possible relationship between calycosin with c-Cbl and TRIM33 should be evaluated in future studies.

PAI-1 is a canonical Wnt/β-catenin signaling pathway-activated gene in kidney tubular epithelial cells [27]; however, its role in CRC cells remains unknown, despite the direct regulation of PAI-1 by BATF2 identified in the present study. We also found that the Wnt/β-catenin signaling was switched to the “Wnt-on” state by calycosin in CRC cells.

## Conclusion

In summary, our study revealed that calycosin induced the upregulation of BATF2, which was antagonized by TGF-β via the STAT3 signaling pathway to inhibit cell growth a through the PI3K pathway by Akt phosphorylation. TGF-β suppressed expression of BAX via the phosphoinositide 3-kinase pathway but induced cell apoptosis .calycosin enhanced the effect of TGF-β on cell apoptosis. It also demonstrated in our research that calycosin inhibited TGF-β-induced migration by up-regulation of BATF2 to regulate PAI-1 expression directly and suppressed TGF-β-induced EMT by retaining β-catenin nuclear localization via the Wnt signaling pathway in human CRC LoVo and HCT116 cells. Thus, we conclude that the upregulation of BATF2 induced by calycosin may be a therapeutic option for CRC.

## Data Availability

Please contact author for data requests.

## Abbreviations

Akt: Protein kinase B
AP-1: Active protein-1
BATF: Basic leucine zipper ATF-like transcription factor 2
BAX: BCL2-associated X
CRC: Colorectal carcinoma
EMT: Epithelial-to-mesenchymal transition
PAI-1: Plasminogen activator inhibitor-1
p-Akt: Phosphorylated protein kinase B
PBS: Phosphate-buffered saline
PCNA: Proliferating cell nuclear antigen
PI3K: Phosphoinositide 3-kinase
p-STAT3-phosphorylated: Signal transducer and activator of transcription 3
siRNA: Small interfering RNA
STAT3: Signal transducer and activator of transcription 3
TGF-β: Transforming growth factor beta
