# Correction to: Calycosin suppresses TGF-β-induced epithelial-to-mesenchymal transition and migration by upregulating BATF2 to target PAI-1 via the Wnt and PI3K/Akt signaling pathways in colorectal cancer cells

**DOI:** 10.1186/s13046-019-1298-5

**Published:** 2019-07-05

**Authors:** Qun Wang, Weijun Lu, Tao Yin, Li Lu

**Affiliations:** 1 Department of Hepatopancreatobiliary Surgery, Hubei Cancer Hospital, Wuhan, Hubei 430079 People’s Republic of China; 2 Department of Medical Oncology, Hubei Cancer Hospital, Tongji Medical College, Huazhong University of Science and Technology, Wuhan, Hubei 430079 People’s Republic of China; 3 Colorectal Cancer Clinical Research Center of Wuhan, Wuhan, Hubei 430079 People’s Republic of China; 4 Colorectal Cancer Clinical Research Center of Hubei Province, Wuhan, Hubei 430079 People’s Republic of China; 5 Department of Gastrointestinal Surgery, Hubei Cancer Hospital, Wuhan, Hubei 430079 People’s Republic of China

**Correction to: J Exp Clin Cancer Res (2019) 38:240**

**https://doi.org/10.1186/s13046-019-1243-7**

In the original publication of this article [1], there is a mistake in Fig. 7. The tags of TGF-beta and calycosin in Western blotting electrophoresis images are reversed.

The corrected Fig. 7 should be:

**Fig. 7:**
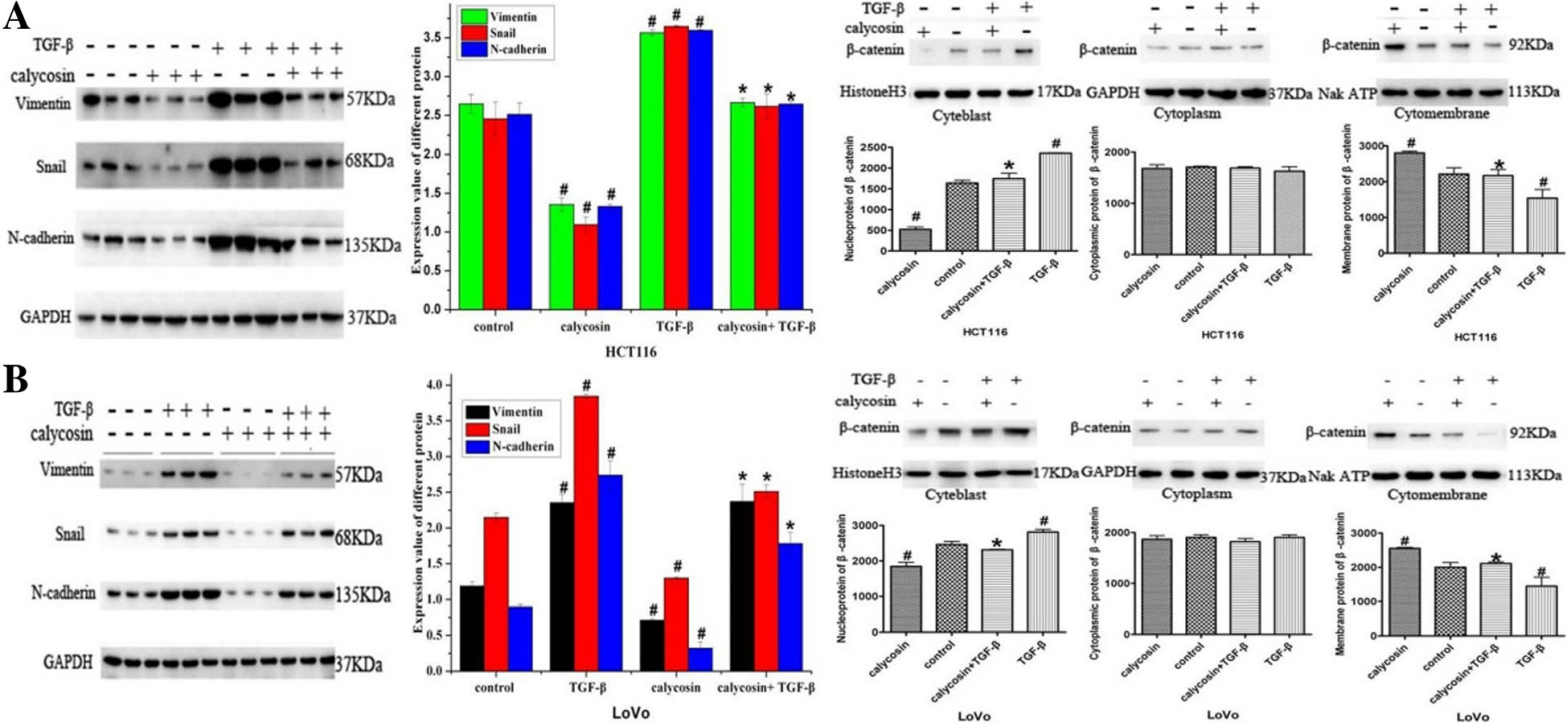
**a** After HCT116 cells were treated with calycosin, TGF-β, or calycosin with TGF-β, western blot analysis was used to determine the protein levels of vimentin, Snail, and N-cadherin and the subcellular distribution of β-catenin protein. **b** After LoVo cells were treated with calycosin, TGF-β, or calycosin with TGF-β, western blot analysis was used to determine the protein levels of vimentin, Snail, and N-cadherin and the subcellular distribution of β-catenin proteins
